# Ultrasonographic Estimation of the Gestational Age Using the Fetal Kidney Length in the Second and Third Trimesters of Pregnancy Among South Indian Antenatal Women: A Cross-Sectional Study

**DOI:** 10.7759/cureus.41172

**Published:** 2023-06-30

**Authors:** Mahima Sophia M, Yuvaraj Maria Francis, Balaji Karunakaran, Sankara Narayanan G

**Affiliations:** 1 Anatomy, Panimalar Medical College Hospital & Research Institute, Chennai, IND; 2 Anatomy, Saveetha Medical College, Saveetha Institute of Medical and Technical Sciences, Chennai, IND

**Keywords:** ultrasonography, head circumference, trimester, gestational age, fetal kidney length

## Abstract

Background and Objective

Accurately estimating the age of the fetus is crucial to prevent morbidity and mortality for both the fetus and mother during the perinatal period. Additionally, it enables early intervention in cases of complicated pregnancies. Multiple parameters are used for the estimation of fetal gestational age (FGA) and the expected date of delivery (EDD), but no single parameter is found to be accurate and standard. This study aims to analyze the fetal kidney length (FKL) as a means to estimate gestational age and investigate the growth pattern of the fetal kidney during the early and late weeks of gestation. Furthermore, it seeks to establish correlations between FKL and gold standard parameters from the 18th week to the 38th week of gestation.

Methodology

This cross-sectional research was carried out after obtaining proper institutional ethical clearance. The pregnant women who came to the gynecology outpatient department (OPD) between 18 and 38 weeks were included in this study after obtaining informed consent. The fetal biometry was measured using the ultrasonographic transducer (3-5 MHz).

Results

The mean FKL exhibited a consistent increase throughout the entire pregnancy, ranging from 16.50 ± 2.10 to 39.20 ± 3.10 mm. The rate of increase in FKL was significant between the early weeks (18-24) of pregnancy, with insignificant growth in other weeks of gestation. The growth of the fetal kidney (length) exhibited a gradual increase from the early weeks to the late weeks of pregnancy, with a consistent growth rate of approximately 1mm per week from 18 to 35 weeks. However, in the final three weeks of pregnancy (36th, 37th, and 38th weeks), the FKL measurements were recorded as 37.90 ± 3.90, 38.90 ± 3.10, and 40.20 ± 3.10 mm, respectively. A positive correlation was noted between the FKL with all standard parameters such as biparietal diameter (BPD), femur length (FL), head circumference (HC), and abdominal circumference (AC).

Conclusions

This study concluded that incorporating FKL alongside standard fetal biometric parameters such as BPD, FL, HC, and AC enhanced the accuracy of calculating FGA and EDD during the early second trimester. Furthermore, it proved beneficial in diagnosing fetal anomalies during early pregnancies.

## Introduction

An accurate fetal gestational age (FGA) is vital to calculate the expected date of delivery (EDD), diagnose congenital anomalies, and provide quality maternal care [1]. Estimations based on menstrual history, quickening, and signs during examination are not always accurate due to variable survival of sperms, irregular menstrual history, uterine pathology, and anomalies [2-6]. Classical fetal biometric parameters such as mean sac diameter, crown-rump length, biparietal diameter (BPD), femur length (FL), head circumference (HC), and abdominal circumference (AC) are used in different periods of gestation. However, all lose reliability as the pregnancy progresses [7,8]. The crown-rump length and the mean sac diameter are most reliable during the first trimester, BPD during the second trimester, and FL during the third trimester [9,10]. The fetal kidney length (FKL) has been proposed as a reliable marker for calculating FGA and EDD, mainly when used in combination with other fetal biometry measurements [1,11,12]. As the kidney starts developing in week 4 of gestation and becomes functional at week 10 or 11, FKL can be measured starting in week 12 [13,14]. However, the use of FKL in estimating age has been reported only in later weeks (20-41) of gestation. This study thus assessed the suitability of FKL as an additional biometric parameter and correlational factor for estimating gestational age in the early second trimester.

## Materials and methods

This cross-sectional study measured fetal biometric parameters in 160 antenatal women at 18 to 38 weeks of gestation who visited the obstetrics department at Saveetha Medical College and Hospital, Thandalam, Chennai, from April 2021 to March 2022. Women whose pregnancies exhibited normal fetal growth with no maternal or fetal complications or comorbidities were enrolled. Ethical approval was sought and obtained from the institutional ethics committee (SMC/IEC/2021/04/12), and after explaining the study in detail, written informed consent was obtained from all study participants. The inclusion criteria for this study comprised all pregnancies without risk factors and with a known last menstrual period, regardless of gravida. Exclusion criteria were classified based on fetal and maternal factors, including fetal anomaly, fetal growth restriction, polycystic kidney disease or other renal anomalies (such as agenesis, hypoplasia, cyst, and hydronephrosis), twinning, late pregnancy, small kidney without peri-renal fat pad, or a gestation period of fewer than 15 weeks. Additionally, hypothyroidism, diabetes, and difficult ultrasonographic imaging due to maternal obesity were also considered as exclusion criteria.

**Figure 1:**
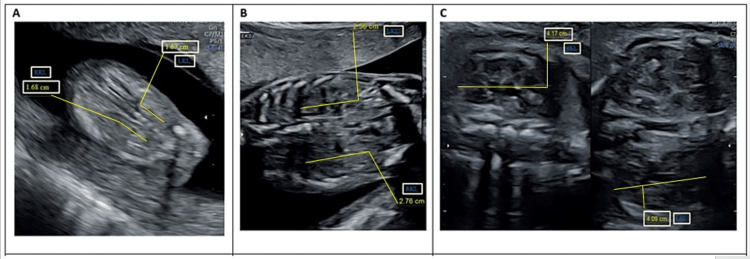
Ultrasound measurements of fetal kidney length at different gestational weeks: (A) 18th week, (B) 25th week, and (C) 37th week.

**Figure 2:**
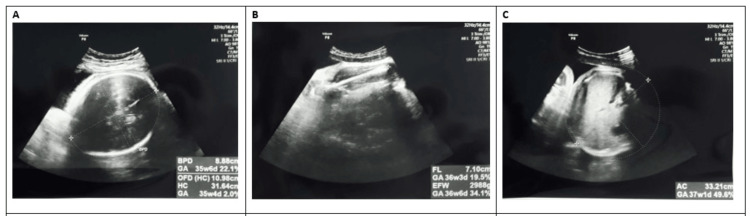
Ultrasound measurement of standard fetal biometry:(A) biparietal diameter and head circumference, (B) femur length, and (C) abdominal circumference.

Fetal biometric parameters (BPD, FL, HC, AC, and right and left FKL) were measured using a Philips HD7 ultrasound transducer at 3-5 MHz (version 2.0.1, KPI Georgia, Tbilisi, GA, USA). After visualizing the four chambers of the heart during ultrasound imaging, the longitudinal axes of both kidneys were noted and, in the same plane, the outer margins of the kidney were used as landmarks to measure kidney length [15]. Measurements were taken three times for each parameter in weeks 18 to 38. Mean values were considered for statistical analyses. Figures 1-2 show the FKL measurements in weeks 15-20, 21-36, and 37 and above.

All statistical analyses were done using IBM SPSS Statistics for Windows, Version 25.0 (IBM Corp., Armonk, NY, USA). FKL and all standard parameters are expressed as mean ± standard deviation. Paired and independent t-tests were used to calculate the level of significance, and a *P*-value <0.05 was considered significant. A Pearson correlation analysis was done to identify correlations between parameters.

## Results

**Figure 3:**
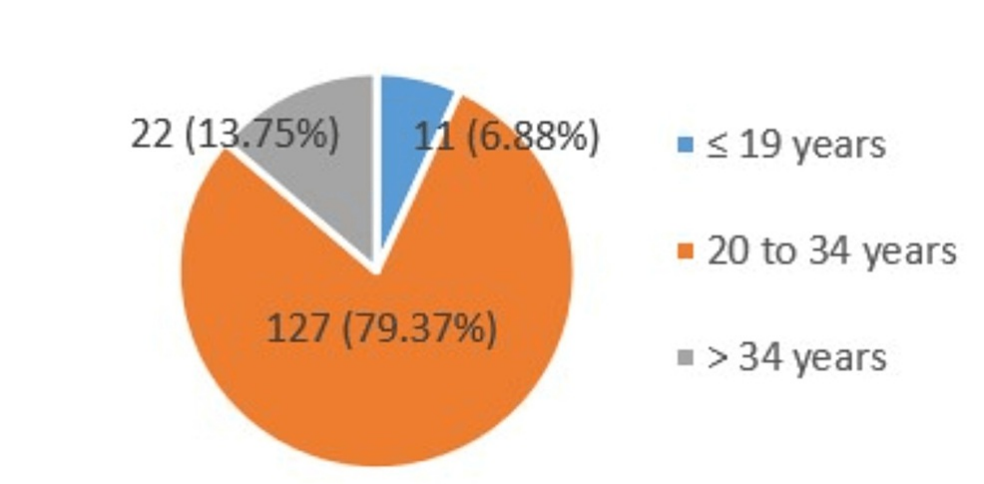
Age distribution of participants.

This study included a total of 160 women who were in the gestational period ranging from weeks 18 to 38 of pregnancy. The majority of women (127/160, 79.37%) were aged 20 to 34 years. This study classifies participants’ pregnancies by parity (primi gravida, second gravida, and multigravida). Most participants had primi gravida (75/160, 46.87%), followed by second gravida (45/160, 28.13%) and multi gravida (40/160, 25%) pregnancies. Figure 3 shows the age distribution (≤19, 20-34, and >34 years) at the time of presentation or checkup.

Table 1 summarizes the fetal biometric measurements (FKL, BPD, FL, HC, and AC) taken in weeks 18 to 38. Mean FKL was 16.50 ± 2.10 to 38.20 ± 3.10 mm. The most observations were collected in gestation weeks 21 to 38 (15 each week) and the least in week 27 (2). As shown in Table 1, FKL increased by 1 mm weekly from weeks 18 (18.50 ± 2.10) to 38 (38.20 ± 3.10). FKL showed constant and correlated growth of 1 mm per week from weeks 18 (18.50 ± 2.10 mm) to 35 (35.30 ± 1.60 mm), with statistically significant increases in weeks 18 to 24 and positive correlations with BPD, FL, HC, and AC.

**Table 1:** Fetal biometric parameters between the weeks of gestation (n = 160). FKL, fetal kidney length; BPD, biparietal diameter; FL, femur length; HC, head circumference; AC, abdominal circumference

S. no.	Gestational age in weeks (*n *= 160)	FKL (mm)	BPD (mm)	FL (mm)	HC (cm)	AC (cm)
1	18 (n1 = 5)	18.50 ± 2.10	42.00 ± 1.30	28.20 ± 1.20	15.82 ± 0.43	13.42 ± 0.62
2	19 (n2 = 6)	19.20 ± 1.90	44.70 ± 1.30	30.40 ± 4.00	17.68 ± 0.90	14.96 ± 0.40
3	20 (n3 = 10)	20.20 ± 2.40	47.40 ± 3.30	32.80 ± 2.70	18.12 ± 1.25	15.84 ± 1.29
4	21 (n4 = 15)	21.50 ± 1.70	51.10 ± 2.20	37.00 ± 2.20	19.27 ± 0.70	16.93 ± 0.92
5	22 (n5 = 9)	22.70 ± 3.80	53.00 ± 3.30	37.90 ± 2.20	19.78 ± 1.21	17.36 ± 1.19
6	23 (n6 = 6)	23.60 ± 2.60	57.40 ± 3.50	41.30 ± 2.00	21.78 ± 0.66	18.52 ± 1.08
7	24 (n7 = 4)	24.30 ± 1.30	60.30 ± 2.80	44.10 ± 1.00	22.46 ± 0.93	20.08 ± 0.39
8	25 (n8 = 7)	25.50 ± 1.10	63.40 ± 4.40	48.20 ± 2.30	24.40 ± 3.68	21.26 ± 0.76
9	26 (n9 = 5)	26.30 ± 1.50	65.80 ± 4.80	51.40 ± 5.30	25.55 ± 4.52	22.21 ± 1.66
10	27 (n10 = 2)	27.40 ± 1.50	70.50 ± 0.70	52.00 ± 0.00	26.20 ± 0.00	22.75 ± 0.78
11	28 (n11 = 6)	28.70 ± 1.20	71.50 ± 3.30	52.60 ± 4.60	26.20 ± 0.99	23.47 ± 0.77
12	29 (n12 = 7)	29.90 ± 1.60	73.40 ± 4.70	55.40 ± 3.30	27.31 ± 1.28	25.64 ± 1.04
13	30 (n13 = 7)	30.40 ± 1.50	75.50 ± 3.80	56.90 ± 2.70	27.90 ± 0.93	25.23 ± 0.55
14	31 (n14 = 11)	31.20 ± 2.00	79.60 ± 2.70	60.50 ± 2.00	29.42 ± 0.73	27.36 ± 1.29
15	32 (n15 = 9)	32.20 ± 1.90	80.70 ± 2.40	63.40 ± 2.20	29.93 ± 0.78	28.04 ± 2.19
16	33 (n16 = 8)	33.50 ± 2.20	81.50 ± 4.20	63.10 ± 3.30	30.40 ± 1.54	28.87 ± 1.40
17	34 (n17 = 7)	34.30 ± 1.50	83.30 ± 1.90	65.60 ± 2.20	31.11 ± 0.50	30.26 ± 1.59
18	35 (n18 = 9)	35.30 ± 1.60	86.90 ± 5.70	67.80 ± 2.80	31.57 ± 1.29	31.31 ± 1.54
19	36 (n19 = 5)	37.90 ± 3.90	84.80 ± 2.50	68.70 ± 1.30	31.73 ± 0.61	31.74 ± 0.84
20	37 (n20 = 7)	38.90 ± 3.10	88.20 ± 2.50	69.50 ± 13.70	32.30 ± 0.60	31.90 ± 0.74
21	38 (n21 = 15)	40.20 ± 3.10	90.60 ±3.30	72.50 ± 2.50	33.01 ± 0.70	33.95 ±1.40

**Figure 4:**
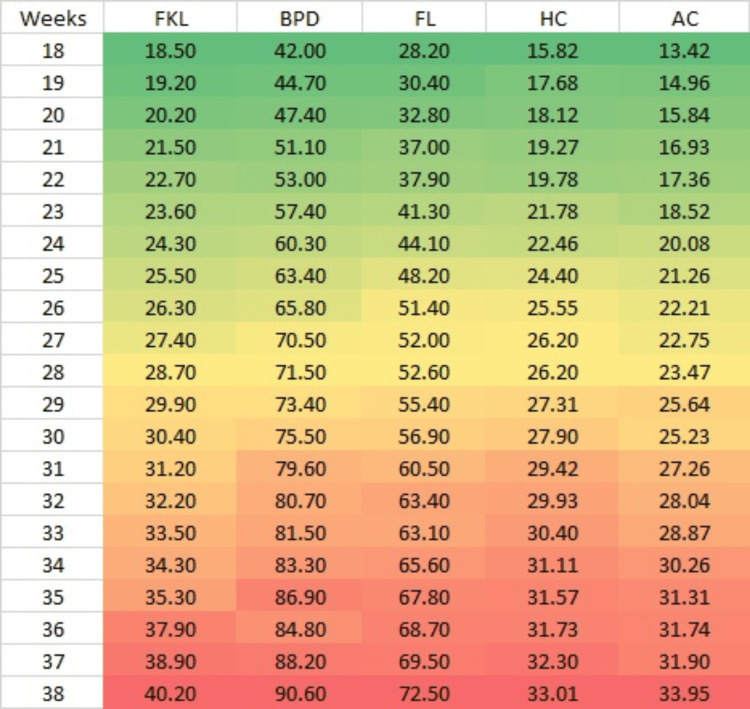
Heatmap comparison of fetal biometric parameters FKL, fetal kidney length; BPD, biparietal diameter; FL, femur length; HC, head circumference; AC, abdominal circumference

Figure 4 shows a heatmap comparing all fetal biometric parameters measured in millimeters. The colors in the heatmap represent the mean values, with red indicating lower values, green indicating higher values, and yellow representing intermediate increasing values. All parameters demonstrate a proportional increase with gestational age. Particularly, FKL and AC exhibit a gradual increase up to 30 weeks of gestation. Therefore, FKL, along with AC and other parameters, can be utilized to determine gestational age effectively.

**Figure 5:**
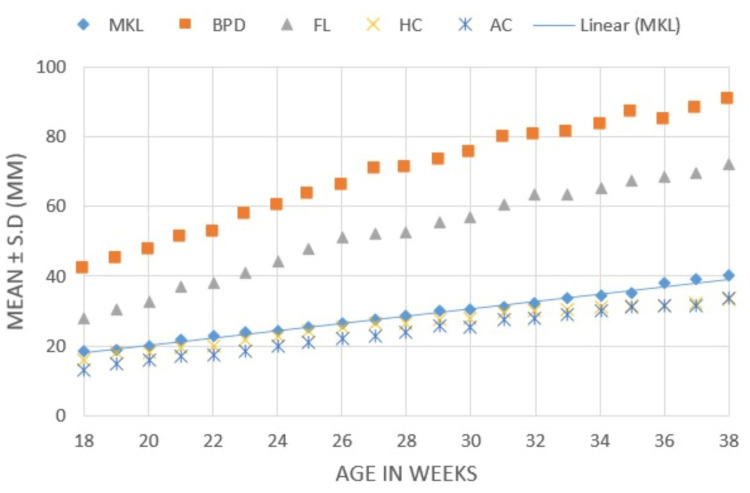
Comparison of sequential growth of fetal biometric parameters with increasing weeks of gestation. MKL, mean kidney length; BPD, biparietal diameter; FL, femur length; HC, head circumference; AC, abdominal circumference

Figure 5 shows the sequential growth of fetal biometric parameters from weeks 18 to 38. We observed that mean FKL increased in a pattern similar to HC and AC but not BPD and FL.

Table 2 shows the comparison of Pearson correlations between FKL and all standard parameters. As shown, changes in FKL were significantly correlated with changes in BPD, FL, HC, and AC (0.951, 0.930, 0.939, and 0.943, respectively) throughout the pregnancy (all *P*-values <0.001, two-tailed).

**Table 2:** Correlation between the mean kidney length and other fetal biometric parameters. *Correlation is significant at the 0.01 level (two-tailed). FKL, fetal kidney length; BPD, biparietal diameter; FL, femur length; HC, head circumference; AC, abdominal circumference

Fetal biometric parameters	Correlations	FKL	BPD	FL	HC	AC
FKL	Pearson correlation	1	0.951^*^	0.930^*^	0.939^*^	0.943^*^
	Sig. (two-tailed)		0.000	0.000	0.000	0.000
	n	160	160	160	160	160
BPD	Pearson correlation	0.951^*^	1	0.956^*^	0.978^*^	0.977^*^
	Sig. (two-tailed)	0.000		0.000	0.000	0.000
	n	160	160	160	160	160
FL	Pearson correlation	0.930^*^	0.956^*^	1	0.943^*^	0.956^*^
	Sig. (two-tailed)	0.000	0.000		0.000	0.000
	n	160	160	160	160	160
HC	Pearson correlation	0.939^*^	0.978^*^	0.943^*^	1	0.963^*^
	Sig. (two-tailed)	0.000	0.000	0.000		0.000
	n	160	160	160	160	160
AC	Pearson correlation	0.943^*^	0.977^*^	0.956^*^	0.963^*^	1
	Sig. (two-tailed)	0.000	0.000	0.000	0.000	
	n	160	160	160	160	160

## Discussion

Several factors can affect fetal growth, including socioeconomic status, nutrition, medical history, genetic diseases, complications during pregnancy or at the time of delivery, parity, age of the mother, and interobserver variability. Failure to account for these factors may help explain the huge variations in the reliability of fetal biometry measurements [16-19]. Ultrasound plays a crucial role in obstetrics to exclude uterine anomalies, measure fetal growth, and predict delivery dates [7]. During organogenesis in the first trimester, the mean sac diameter and crown-rump length are the standard parameters used to calculate the gestational age of the fetus [9,10,20]. After organ maturation, various other measurements (e.g., BPD, FL, AC, HC, and FKL) have been used to estimate the date of delivery and fetal growth [21]. A study by Karki et al. reported that AC and BPD are the best and least correlated parameters, respectively, in the second trimester, and HC and FL are most suitable in the third trimester [20]. Konje et al. found that FKL, FL, and AC are the best and least correlated parameters, respectively, to calculate gestational age in weeks 24 to 38 [1]. Uterine pathologies or anomalies can further complicate these calculations [5]. Recent studies showed that FKL may be a useful biometric parameter to calculate the date of delivery [22-25].

In this study, FKL was measured in weeks 18 to 38 and increased by 1 mm each week (18.50 ± 2.10 mm in week 18 to 35.30 ± 1.60 mm in week 35), exhibiting statistically significant increases in weeks 18 to 24, in a manner positively correlated with standard fetal biometry parameters (BPD, FL, HC, and AC). Previous studies have found that FKL measurements (mm) align with the gestational week of pregnancy in the first trimester and early second trimester (e.g., 22 mm at 22 week) [12,26-30]. Konje et al. noted significantly higher FKL in alternate weeks of gestation starting in week 24 compared to this study [1], whereas Kansaria and Parulekar and Akintomide and Efanga reported lower growth rates [23,31]. Similar to the current findings, Edevbie et al. reported corresponding FKL measurements with weeks of gestation (i.e., from weeks 20 to 36), with no coinciding FKL values in the last weeks of gestation [22]. Ugur et al. reported that FKL is positively correlated with gestational age and is an accurate parameter for predicting the date of delivery in late pregnancy [32]. Abbas et al. measured FKL using ultrasound and manual methods and found that ultrasound is more accurate. They further reported a positive correlation between FKL and gestational age [33]. In contrast to this study, Cohen et al. reported that FKL was not correlated with gestational age in calculating the EDD (*r *= 0.00) and that accurate FKL was needed to exclude abnormal fetal growth [34]. Overall, these findings indicated that FKL measurements in the second trimester may be helpful to calculate delivery dates and identify fetal anomalies. Table 3 summarizes findings from the literature showing how FKL (mm) coincides with gestation age (weeks).

**Table 3:** Comparing fetal kidney lengths (mm) and gestational age (weeks). FKL, fetal kidney length; WOG, weeks of gestation

Mean FKL												
WOG	This study	Joshi et al. [27]	Saxena et al. [26]	Konje et al. [1]	Ansari et al. [28]	Edevbie and Akhigbe [22]	Ugwuanyi et al. [29]	Kansaria and Parulekar [23]	Shivalingaiah et al. [30]	Yusuf et al. [12]	Abbas et al. [33]	Akintomide and Efanga [31]
16	-	-	-	-	16	-	-	-	-	-	15.5	-
17	-	-	-	-	16	-	-	-	-	-	17.8	-
18	18.50 ± 2.10	-	-	-	18	-	-	-	-	-	21.8	-
19	19.20 ± 1.90	-	-	-	18	-	-	-	-	-	22.2	-
20	20.20 ± 2.40	20.4	-	-	19	20.87	20	-	-	-	24.8	19.2
21	21.50 ± 1.70	21.6	-	-	21	20.96	21	-	-	-	25.5	20
22	22.70 ± 3.80	22.5	-	-	22	22.27	22	-	-	-	26.6	21.3
23	23.60 ± 2.60	23.4	-	-	22	23.79	23	-	-	-	29.4	21.6
24	24.30 ± 1.30	24.6	-	24.2	24	25.33	24	23.87	24.1	-	30.4	23
25	25.50 ± 1.10	25.1	-	-	25	25.97	25	-	-	-	32.5	24.1
26	26.30 ± 1.50	25.9	-	26.3	26	27.47	32	25.23	-	-	33.8	25.2
27	27.40 ± 1.50	26.4	-	-	27	27.54	33	-	-	-	34.4	27.4
28	28.70 ± 1.20	28.1	28	29	28	29.46	34	26.98	28.2	-	34.5	29.4
29	29.90 ± 1.60	29	29	-	29	30.34	35	-	-	-	35.9	29.9
30	30.40 ± 1.50	30.4	29.67	30.9	31	31.83	34	29.03	-	-	37.8	30.4
31	31.20 ± 2.00	31.4	30.28	-	32	31.87	35	-	-	31.4	37.8	31
32	32.20 ± 1.90	31.8	32	33.2	32	33.60	36	30.80	32.8	32	40.8	32.4
33	33.50 ± 2.20	33.3	32.88	-	32	34.31	34	-	-	33	41.3	33.8
34	34.30 ± 1.50	34.7	33.44	35.0	33	35.06	40	32.51	-	34.2	41.9	34.5
35	35.30 ± 1.60	36	34.68	-	34	35.86	40	-	-	35.1	41.9	-
36	37.90 ± 3.90	35.3	35.76	38.2	35	36.86	40	34.26	36.5	35.9	42	-
37	38.90 ± 3.10	37.4	36.76	-	36	38.18	41	-	-	36.9	42.1	-
38	40.20 ± 3.10	-	37.40	40.1	37	38.77	42	36.25	-	37	-	-
39	-	-	38	-	38	39.40	42	-	-	39.3	-	-
40	-	-	40	-	39.5	40.32	42	-	-	40.5	-	-
41	-	-	-	-	-	41.41	-	-	-	-	-	-

Kansaria and Parulekar reported that FKL was highly accurate in predicting the date of delivery, with a standard error of 9.17 days, and AC was least accurate, with a standard error of 11.14 days [23], and these findings have been corroborated in another study [25]. Shivalingaiah et al. found that FKL was highly correlated with all standard fetal biometric parameters (*P*-value < 0.05, *r*2 = 0.85 to 0.98) except AC and that FL at week 24 and BPD at week 36 were not correlated with FKL [30]. Edevbie and Akhigbe stated that gestational age calculated by FKL is linearly correlated with gestational age calculated by BPD, FL, AC, and HC [22]. Cohen et al. found a strong correlation with gestational age, with the average of three fetal biometric parameters (BPD, FL, and AC) and a significant difference between FKL and gestational age throughout gestation [34]. Similarly, Akintomide and Efanga suggested that HC and FL were positively correlated with FKL in calculating the EDD [31]. Table 4 summarizes results from the different studies using FKL to assess gestational age.

**Table 4:** Comparing the periods of gestation with similar FKL (mm) and gestational age (weeks). FKL, fetal kidney length

S. no.	Author	Year	Country	Periods of gestation in which the FKL (mm) corresponds with the gestational age (weeks)
1.	Present study	2022	Tamil Nadu, India	18-35 weeks
2.	Joshi et al. [27]	2021	Nepal	20-25, 28-31, 33, 34, and 37 weeks
3.	Saxena et al. [26]	2016	Rajasthan, India	28, 29, and 32 weeks
4.	Konje et al. [1]	2002	UK	24, 26, and 30 weeks
5.	Ansari et al. [28]	1997	Bangladesh	16, 18, 21, 22, and 24-29 weeks
6.	Edevbie and Akhigbe [22]	2018	Nigeria	20, 22, 23, 25, 27-29, 31, 35, 36, and 38-41 weeks
7.	Ugwuanyi et al. [29]	2018	Nigera	20-25 weeks
8.	Kansaria and Parulekar [23]	2009	Mumbai, India	No weeks are similar with FKL in millimeters
9.	Shivalingaiah et al. [30]	2014	Karnataka, India	24, 28, 32, and 36 weeks (observed every four weeks, all similar)
10.	Yusuf et al. [12]	2007	Bangladesh	31-35, 39, and 40 weeks
11.	Abbas et al. [33]	2012	Pakistan	17 weeks
12.	Akintomide and Efanga [31]	2022	Nigeria	27 and 29-34 weeks

In this study, FKL was significantly positively correlated with standard fetal biometric parameters BPD, FL, HC, and AC (*P*-value = 0.001). The Pearson correlations for FKL were 0.951, 0.930, 0.939, and 0.943 with BPD, FL, HC, and AC, respectively. As mentioned, the number of weeks in the second trimester coincides closely with FKL in millimeters. Thus, FKL may play an important role in assessing FGA, EDD, and fetal anomalies in early pregnancy.

## Conclusions

An accurate FGA helps obstetricians calculate the date of delivery, diagnose congenital anomalies, and provide quality maternal care. In addition to classical fetal biometric parameters, FKL is a potentially useful parameter during ultrasound and antenatal checkups. In this study, FKL in the second trimester accurately predicted the EDD. However, it is important to note that the study has limitations, as it relied on prevalent data and lacked regular follow-up of antenatal cases. To address this limitation, future research should incorporate the same methodology with regular follow-up of antenatal cases. Furthermore, additional studies are necessary to explore other fetal biometric data that can enhance obstetric care and improve patient outcomes.
